# Force and Temperature Characterization of a Novel Fiber Bragg Grating Overhead Line Sensor

**DOI:** 10.3390/s25247425

**Published:** 2025-12-06

**Authors:** Grzegorz Fusiek, Pawel Niewczas

**Affiliations:** Department of Electronic and Electrical Engineering, University of Strathclyde, Glasgow G1 1XQ, UK; p.niewczas@strath.ac.uk

**Keywords:** fiber Bragg gratings, optical sag sensor, high-temperature low-sag conductors, power grids

## Abstract

This paper presents the characterization of a new optical sensor designed for monitoring overhead power lines (OHLs) by determining key mechanical parameters of electrical conductors. The device employs fiber Bragg gratings (FBGs) written into a metal-coated fiber and enclosed within a Kovar^®^ capillary tube. Its epoxy-free design provides robust hermetic protection for the FBGs, enabling reliable performance with both conventional low-temperature and high-temperature low-sag (HTLS) conductors. The sensor configuration enables direct measurements of conductor strain and temperature, as well as indirect estimation of sag and related mechanical quantities such as tension and stress. Laboratory tests were carried out over a temperature range of 30 °C to 200 °C and for applied forces up to 2 kN. The experimentally determined sensitivities were about 0.4 nm/kN for force and 27 pm/°C for temperature. The device endured ten successive thermal cycles between 30 °C and 200 °C, maintaining its force sensitivity within 20% variation throughout the tests. These results confirm that the developed sensor can simultaneously track temperature and mechanical load across the investigated temperature range, demonstrating its potential for HTLS conductor monitoring in power transmission networks.

## 1. Introduction

To improve the power transfer capability, efficiency, and reliability of electrical transmission networks, dynamic line rating (DLR) systems have been developed [1,2]. These systems estimate the real-time current-carrying capacity of a line based on environmental conditions and key conductor parameters such as temperature and sag [3,4]. In parallel with the deployment of DLR systems, several types of conductors have been introduced, including hard-drawn copper and aluminum conductors as well as high-temperature low-sag (HTLS) designs capable of continuous operation at 180–200 °C and short-term operation up to 250 °C, to further enhance grid capacity [4].

Ensuring the operational safety of the electrical grid requires continuous monitoring of overhead line (OHL) conductors, encompassing both electrical and mechanical parameters. This can be achieved either by integrating meteorological model data with local weather sensor measurements to estimate the line’s real-time condition and capacity, or by directly measuring its physical parameters [1,2]. A variety of established technologies have been applied to monitor overhead line (OHL) parameters, including current, temperature, sag, humidity, illumination, bending, and vibration, using electronic, optoelectronic, or optical sensors [3,4,5,6,7,8]. However, many of these methods have limited suitability in harsh temperature environments or for long-distance applications. Wireless electronic sensors, in particular, can be unsuitable for such conditions, and their use is often restricted by network operators because of cybersecurity concerns.

Among the technologies mentioned above, optical sensors, and especially fiber Bragg gratings (FBGs), appear to offer significant advantages in addressing cybersecurity challenges while facilitating multiplexing and remote sensing under harsh environmental conditions and at high-temperature applications [9,10,11]. Sag, temperature, and vibration sensors based on fiber Bragg grating (FBG) technology, as previously demonstrated by the authors [12], can be integrated with FBG-based voltage and current sensors to create a comprehensive monitoring system. Such a configuration enables simultaneous real-time measurement of electrical and mechanical parameters of overhead lines across an extended area.

Since accurate measurement of OHL parameters directly impacts the accuracy of line capacity estimation, the authors recently improved their previous sag sensors by proposing a novel concept design [13]. This new design has the potential to enhance conductor temperature measurement by improving the thermal contact between the FBG temperature and strain sensors and the conductor surface.

This paper focuses on demonstrating the proof of concept for the newly developed sag sensor, describing its fabrication process and initial characterization in terms of force and temperature response. The sensor was tested experimentally in the laboratory over a temperature range of 30 °C to 200 °C and for applied loads up to 2 kN. It maintained structural integrity throughout ten consecutive thermal cycles within this range, with variations in force sensitivity remaining below 20%. The findings confirm that the sensor can reliably measure both mechanical load and temperature under the tested conditions, indicating its suitability for monitoring HTLS conductors.

## 2. Optical Sag Sensor

### 2.1. Fiber Bragg Grating Strain and Temperature Sensor Array

**Figure 1:**
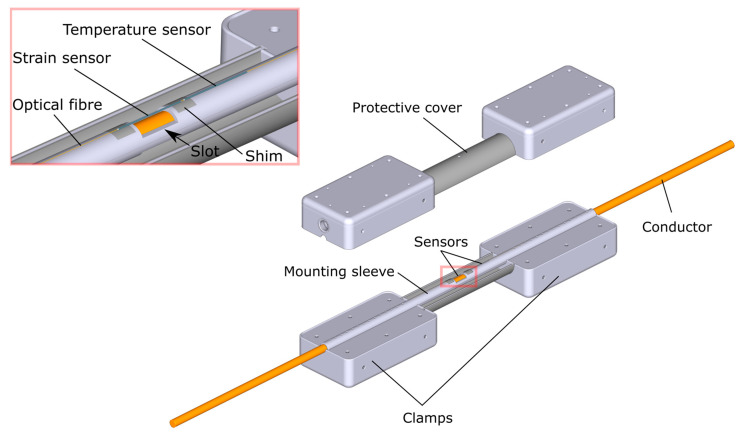
Illustrative diagram of a novel FBG sag sensor clamped on the HDC conductor.

The sag sensor was designed for installation on a 10.5 mm diameter hard-drawn copper (HDC) conductor and incorporates two FBG-based elements to measure conductor strain and temperature [13]. Each element consists of a 10 mm long FBG inscribed within a 15 mm section of copper-coated fiber (Cu1300) [14], which is housed inside a 35 mm long Kovar^®^ capillary (Future Alloys, Neath, UK) with an inner diameter of 200 µm and an outer diameter of 700 µm. The assembly is brazed using a silver-based paste at approximately 620 °C, with heat applied via an induction heating system [15]. The temperature sensor is attached at one end to a strain sensor shim, mechanically isolating it from the strain measurement. This arrangement allows the temperature sensor to provide both independent absolute temperature readings and accurate compensation for temperature effects in the strain sensor, which is necessary because FBGs respond to both strain and temperature variations [16]. An illustrative diagram of a novel FBG sag sensor clamped onto the HDC conductor is shown in Figure 1.

The procedure for encapsulating the strain and temperature FBGs inside Kovar capillaries has previously been presented in [17] and is briefly described below.

A copper-coated fiber with two FBGs inscribed into 15 mm bare sections was threaded through two 35 mm Kovar capillaries, with the capillaries centered over the FBGs. Stainless steel shims, 0.1 mm thick and cut into 15 × 20 mm sections, were positioned at the ends of the strain-sensing FBG capillary. The FBGs were separated by a distance of 36 mm physically and 4 nm spectrally. The FBG pair, along with capillaries and shims, was arranged on a quartz stage and held in place with heat-resistant tape. A silver-based solder paste was applied over the ends of the capillaries where they were in contact with the shims. To minimize oxidation and improve the flow of the brazing material, flux powder was applied to the shims and the joints. The quartz plate with the shims and capillaries was placed on the stand in the induction brazing machine. The fiber was held in clamps attached to the micro-positioning stages, and a small amount of tension was applied to prevent any bending of the fiber during the brazing process. After securing the FBG setup, the fiber end was spliced to a pigtail to monitor wavelength shifts during the brazing process using a SmartScan interrogator (Smart Fibres, Bracknell, UK).

The induction brazing machine was used to minimize heating time and provide a strong mechanical joint while avoiding overheating or damage to the FBG. To prevent spectral overlap between the strain and temperature FBG peaks, the joint at the end of the strain sensor, furthest from the temperature FBG, was brazed first. After the components were allowed to cool, the shim connecting the strain and temperature FBG capillaries was brazed.

After completing the strain sensor, the other end of the temperature FBG capillary was sealed. A quartz plate with two channels and alumina blocks with grooves held the Kovar capillary in place. A silver-based paste was applied at the end of the capillary, and two steel quarter cylinders with a slot hole cut into them were used as a heat concentrator, facilitating a solid joint between the capillary and fiber.

After this process, the joints and the shims were cleaned. The components were soaked in malt vinegar for a few hours and then rinsed with hot water. The shims were wet sanded to prepare them for spot welding using fine-grit sandpaper.

### 2.2. Sag Sensor Assembly

**Figure 2:**
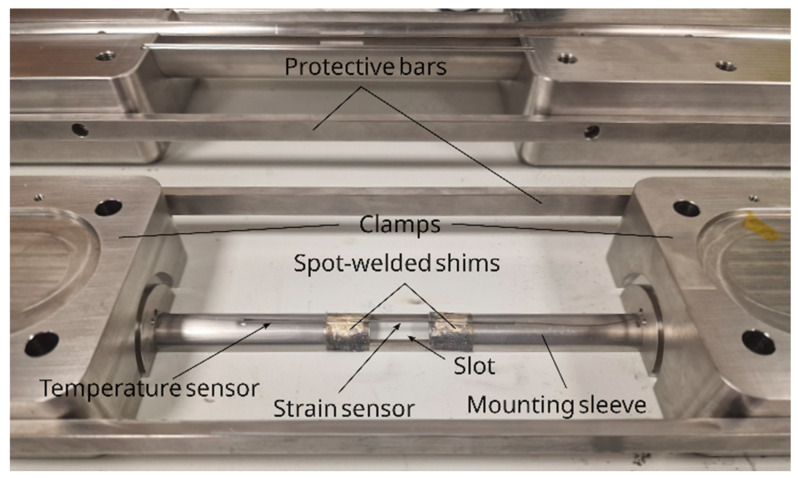
The strain and temperature sensor pair welded to the mounting sleeve at both shims after prestressing the strain sensor.

Firstly, the top and bottom parts of the sag sensor were assembled. This involved fixing the mounting sleeve halves to the corresponding clamps with M2 screws and securing the clamps with the protective bars, as shown in Figure 2. The strain and temperature sensor pair was then positioned across the slot in the mounting sleeve, and the shim between the temperature and strain sensors was spot-welded to the sleeve. To ensure sufficient spectral spacing between temperature and strain sensing FBGs during operation, the strain sensor was slightly prestressed (approximately 0.5 µm/m) with a bespoke prestressing tool, and the other shim was then spot-welded to the sleeve. The process of prestressing the strain sensor was monitored online using the SmartScan FBG interrogator.

**Figure 3:**
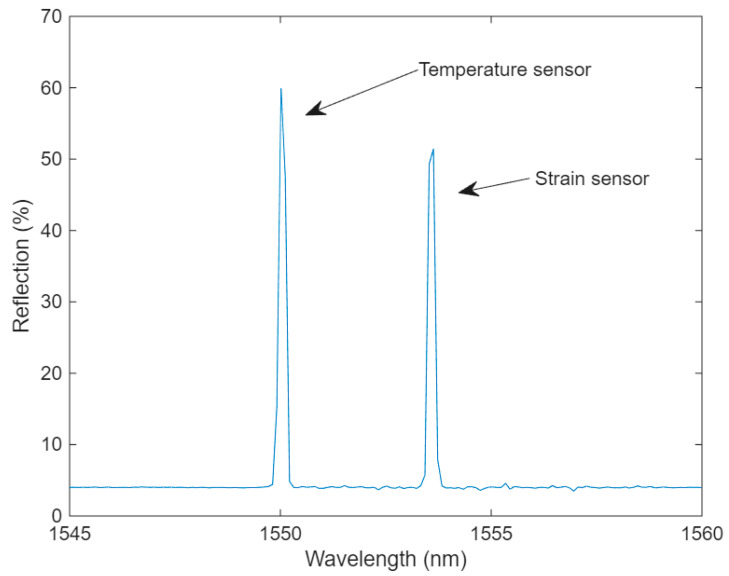
Sag sensor spectrum after prestressing and spot welding to the mounting sleeve.

The temperature and strain sensor array after welding to the mounting sleeve is shown in Figure 2, and a spectrum of the sensors is presented in Figure 3.

It should be noted that for characterization, the sensor was installed on an HDC rod instead of an HDC conductor because of limitations of the tensioning machine. Using a rod was necessary due to the restricted space between the machine grips inside the oven and the difficulty of properly securing the conductor in these grips. The impact of using the HDC rod instead of the actual conductor on the test results is expected to be marginal, as the physical and mechanical parameters of the rod and conductor are matched. However, the cross-sectional area of the rod is slightly larger than that of the conductor with the same diameter (86 mm^2^ vs. 67 mm^2^). Therefore, for real-world applications, the sensor must be calibrated on the same type of conductor on which it will ultimately be installed.

## 3. Results

### 3.1. Experimental Setup for Sensor Characterization

**Figure 4:**
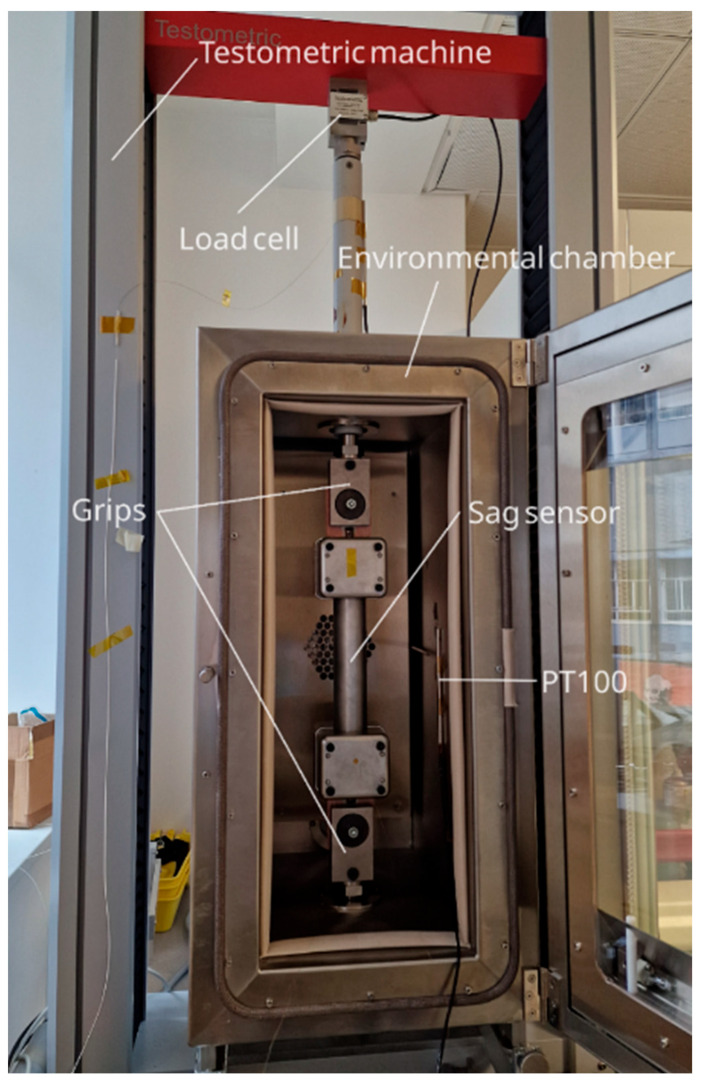
Sag sensor setup in the Testometric tensioning machine.

After assembly, the sag sensor was mounted on a 10.5 mm diameter HDC rod, serving as a surrogate for the HDC conductor, and placed in a 10 kN tensioning machine (M350-10CT Testometric, Rochdale, UK). This machine allows for precise control of load ranges and rates, with speeds from 0.00001 to 2000 mm/min, and provides a load measurement accuracy of ±0.5% down to 1/1000th of the load cell capacity. The machine also features an environmental chamber capable of cycling the sensor at temperatures between 30 and 200 °C. For performance evaluation, the copper rod with the attached sensor was secured in the machine grips, as illustrated in Figure 4, and protective bars were removed prior to testing.

Temperature inside the chamber was controlled via the machine’s integrated temperature controller and monitored using a 4-channel PT-104 temperature logger (Pico Technology) with ±0.01 °C accuracy, as well as a PT100 platinum resistance thermometer (PRT) covering −50 °C to +250 °C with an accuracy of ±0.03 °C.

During the force and temperature characterization, the applied load varied between 0.5 and 2 kN. The machine crosshead increased the force at a rate of 1 mm/min, held it constant for 2 min, and then decreased it back to 0.5 kN at the same rate. Force cycling was performed at temperatures of 30 °C, 40 °C, 80 °C, 120 °C, 160 °C, and 200 °C. At each temperature, the chamber was allowed to stabilize for one hour before measurements commenced. Three force cycles were conducted at each temperature level, with readings recorded by the machine software. Simultaneously, shifts in the FBG center wavelength (FBG CW) of the sag sensor were captured by the FBG I-MON 256 USB interrogator (Ibsen Photonics, Farum, Denmark) at 1 kHz and logged every second, alongside temperature and load cell data. The results of this characterization are presented below.

### 3.2. Characterization of Force and Temperature Response

**Figure 5:**
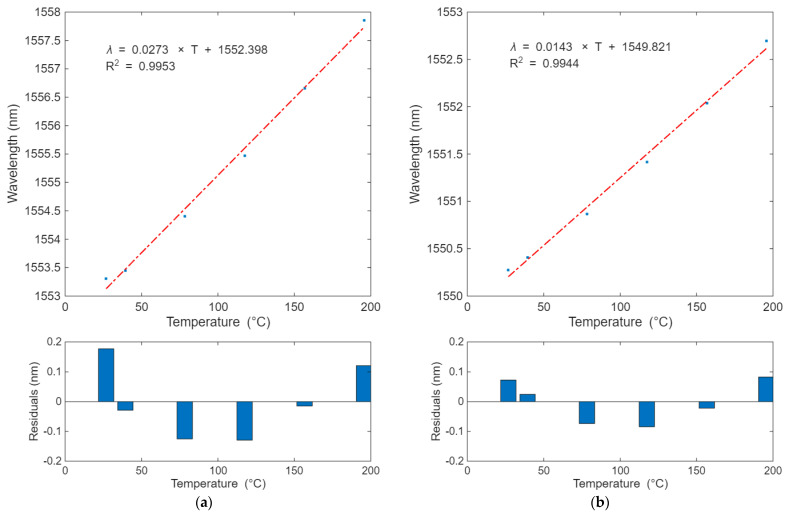
Sag sensor temperature characterization: (**a**) strain sensor temperature response; (**b**) temperature sensor temperature response.

The temperature response of the sag sensor was evaluated using readings from the PT100 probe and the PT-104 logger. The behavior of the strain and temperature sensors over the most stable and uniform regions is presented in Figure 5a,b, respectively.

Based on a linear fitting into the experimental data, the strain sensor’s temperature sensitivity was estimated at 27.3 pm/°C, while the temperature sensor’s temperature sensitivity was determined to be 14.3 pm/°C. The linear fitting into each sensor response was performed with a 95% confidence level, and the relevant coefficients of determination (R^2^) were 0.9953 and 0.9944, respectively.

**Figure 6:**
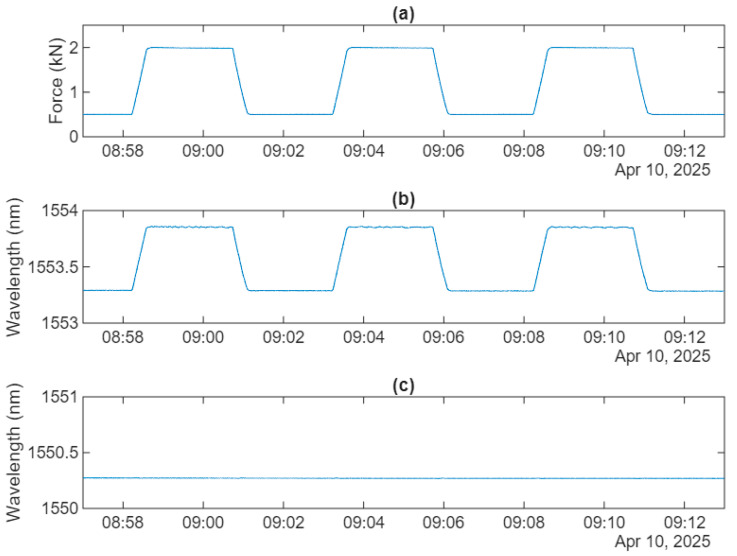
Response of the test sag sensor to force cycling at 30 °C: (**a**) load cell; (**b**) strain sensor; (**c**) temperature sensor.

Figure 6 illustrates examples of the applied force, varying from 0.5 kN to 2 kN, along with the corresponding strain and temperature FBG wavelength signals recorded at 30 °C over three cycles. During characterization, the force was gradually increased from 0.5 kN to 2 kN and then decreased back to 0.5 kN, with each cycle lasting about five minutes.

**Figure 7:**
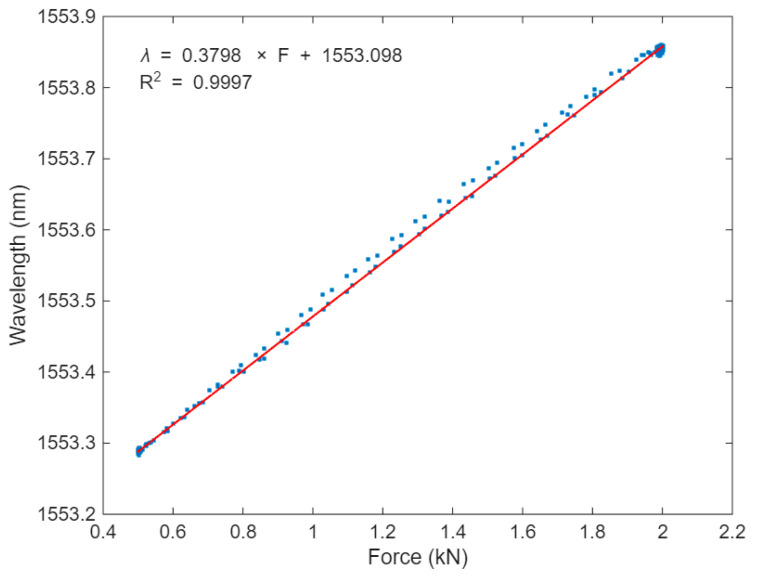
Force response of the sag sensor at 30 °C.

As presented in Figure 6, the temperature sensor remains unresponsive to force, while the strain sensor exhibits a peak shift of approximately 0.5 nm for a force change of 1.5 kN. Based on this, the sag sensor’s force sensitivity, S_F_, at 30 °C was estimated at around 0.38 nm/kN, as shown in Figure 7. The linear fitting was performed on the data acquired from two cycles of the applied force, encompassing both hysteresis and nonlinearity in the sensor response. The coefficient of determination (R^2^) was 0.9997 in this case.

**Figure 8:**
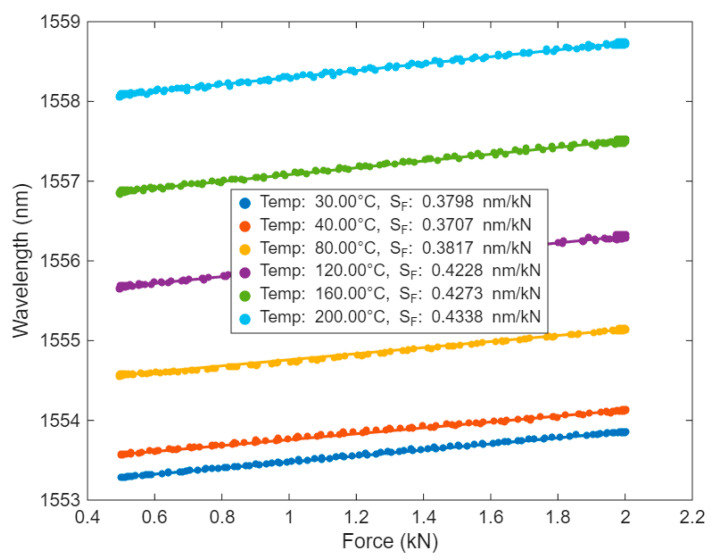
Force response of the sag sensor over 30–200 °C.

Force characterization was also performed at temperatures between 30 °C and 200 °C, and the sensor’s responses are presented in Figure 8. The linear fitting was performed in the same way as specified above.

**Figure 9:**
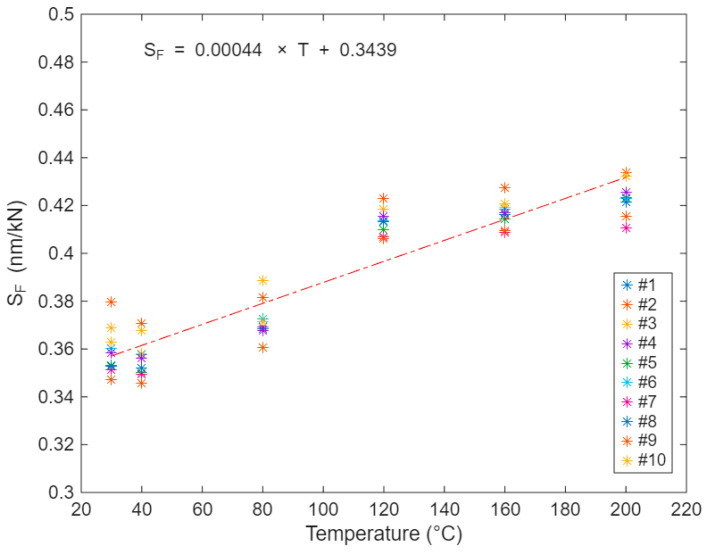
Sag sensor force sensitivity for 10 consecutive runs at temperatures between 30 °C and 200 °C.

The average force sensitivity of the strain sensor over this temperature range was 0.403 ± 0.028 nm/kN (mean ± standard deviation), indicating stable performance across varying temperatures. The sensor endured ten consecutive thermal cycles between 30 °C and 200 °C, with variations in force sensitivity remaining below 20% throughout the tested range, as illustrated in Figure 9.

As can be seen in Figure 9, the force sensitivity scattered randomly over the investigated temperature range within the 10 runs, with no apparent drift in the data.

## 4. Discussion

As mentioned in Section 3.1, the load cell used had an overall accuracy of 0.5% of the reading, while its thermal drift was specified by the manufacturer as less than 0.005% per applied load per °C. During the sensor characterization at 200 °C in the chamber, the temperature around the load cell reached 60 °C, resulting in a maximum force drift of 0.2% at a 2 kN load and at 200 °C in the chamber. However, the maximum operating temperature of the cell is 80 °C, and it features temperature compensation to maintain accuracy up to 70 °C, according to the manufacturer’s specifications. The standard uncertainty in force measurement at a load of 2 kN was estimated to be below 0.3%, assuming a uniform distribution of errors over the load cell’s measurement range.

The combined standard uncertainty in temperature measurement, arising from the temperature logger and probe, was estimated to be 0.0182 °C, which was lower than the corresponding uncertainties due to the interrogator system.

The combined uncertainty in wavelength measurement, resulting from the interrogator wavelength fit resolution (<0.5 pm standard deviation) and wavelength repeatability (3 pm, peak-to-peak for all polarization states), was estimated to be 1.8 pm. Since the interrogator unit was thermally compensated and maintained in a controlled environment at a constant ambient temperature of 21 °C, wavelength drifts due to temperature were neither observed nor considered in the uncertainty calculations.

Owing to the differing temperature sensitivities of the strain and temperature sensors, their combined standard uncertainties in temperature measurements were estimated at 0.06 °C and 0.12 °C, respectively, when read by the I-MON interrogator. The overall uncertainty in force measurements, considering both the load cell and interrogator, was estimated to be below 1%.

Despite the slight force sensitivity variation (below 20%) across the considered temperature range, the sensor remains fully viable for practical field deployment provided that appropriate compensation measures are implemented. In real applications, this issue is best addressed by establishing a multi-temperature calibration framework, where the force–wavelength response is characterized at several controlled temperatures spanning the expected operating range. The temperature FBG integrated within the sensor assembly can then be used to dynamically select or interpolate the appropriate calibration factor during operation. A temperature-dependent calibration model, for example, a polynomial or piecewise-linear function, can be embedded in the acquisition software to automate this compensation. Through this approach, the influence of temperature-induced sensitivity changes can typically be reduced to a residual error well within utility requirements, thereby enabling reliable tension and sag estimation for HTLS and conventional conductors under varying environmental and loading conditions.

## 5. Conclusions

This paper has presented the method of fabrication and characterization of a novel optical sag sensor incorporating FBG-based strain and temperature elements. The sensor was mounted on an HDC rod rather than directly on a conductor and was tested for its force and temperature response over the range of 30 and 200 °C. The temperature sensitivities of the strain and temperature sensors were measured to be 27.3 pm/°C and 14.3 pm/°C, respectively. The average force sensitivity of the strain sensor across the tested temperature range was 0.403 ± 0.028 nm/kN, indicating good stability. The sensor withstood ten consecutive test runs between 30 °C to 200 °C, with variation in force sensitivity below 20%, demonstrating its robustness and reliability under thermal cycling. Based on these characterization results, the sensor appears well-suited for monitoring mechanical parameters of HTLS conductors. Future work will investigate the sensor’s capability to monitor conductor sag and fatigue under real operating conditions. In addition, the long-term stability of the sensor at elevated temperatures will be evaluated in laboratory conditions.

## Data Availability

All data underpinning this publication are openly available from the University of Strathclyde Knowledge Base at https://doi.org/10.15129/60941ccd-c97a-403a-9951-2ba7c77b51a3 (accessed on 23 November 2025).

## References

[1] (2007). Technical Brochure 332: Fatigue Endurance Capability of Conductor/Clamp Systems-Update of Present Knowledge.

[2] (2007). Technical Brochure 324: Sag-Tension Calculation Methods for Overhead Lines.

[3] de Nazare F.V.B., Werneck M.M. (2012). Hybrid Optoelectronic Sensor for Current and Temperature Monitoring in Overhead Transmission Lines. IEEE Sens. J..

[4] Wang H., Han S., Lv L.-J., Jin L.-J. (2017). Transmission Line Sag Measurement Based on Single Aerial Image. Proceedings of the 2017 24th International Conference on Mechatronics and Machine Vision in Practice (M2VIP).

[5] Wydra M., Kisala P., Harasim D., Kacejko P. (2018). Overhead Transmission Line Sag Estimation Using a Simple Optomechanical System with Chirped Fiber Bragg Gratings. Part 1: Preliminary Measurements. Sensors.

[6] Hsu T.S., Chiu H.C., Yang Y.C., Tseng C.Y., Lin M.J., Wang J.C., Jiang J.A. (2019). An IoT-Based Sag Monitoring System for Overhead Transmission Lines. Proceedings of the 2019 IEEE PES GTD Grand International Conference and Exposition Asia (GTD Asia).

[7] Kopsidas K., Rahman S.A., AlAqil M.A., Rolfo S. (2023). Advancing OHL Rating Calculations: Modeling Mixed-Convective Cooling and Conductor Geometry. IEEE Trans. Power Deliv..

[8] Chen Y., Ding X. (2023). A Survey of Sag Monitoring Methods for Power Grid Transmission Lines. IET Gener. Transm. Distrib..

[9] Dedyulin S., Ahmed Z., Machin G. (2022). Emerging Technologies in the Field of Thermometry. Meas. Sci. Technol..

[10] Schukar V.G., Kadoke D., Kusche N., Münzenberger S., Gründer K.P., Habel W.R. (2012). Validation and Qualification of Surface-Applied Fibre Optic Strain Sensors Using Application-Independent Optical Techniques. Meas. Sci. Technol..

[11] Lindner M., Bernard D., Heilmeier F., Jakobi M., Volk W., Koch A.W., Roths J. (2020). Transition from Purely Elastic to Viscoelastic Behavior of Silica Optical Fibers at High Temperatures Characterized Using Regenerated Bragg Gratings. Opt. Express.

[12] Fusiek G., Niewczas P. Design of an Optical Sensor with Varied Sensitivities for Overhead Line Sag, Temperature and Vibration Monitoring. Proceedings of the I2MTC 2022-2022 IEEE International Instrumentation and Measurement Technology Conference.

[13] Marston J., Fusiek G., Niewczas P. (2024). Design of a Novel Optical Overhead Line Monitoring Sensor. IEEE Sens. Lett..

[14] IVG Fiber Copper-Coated Single Mode Fiber, Cu1300. https://www.ivgfiber.com/datasheet_singlemode.pdf.

[15] Niewczas P., Fusiek G. Induction Heating Assisted Optical Fiber Bonding and Sealing Technique. Proceedings of the SPIE—The International Society for Optical Engineering.

[16] Rao Y.-J. (1997). In-Fibre Bragg Grating Sensors. Meas. Sci. Technol..

[17] Fusiek G., Niewczas P., Johnston M. (2014). Metal-Packaged Fiber Bragg Gratings for Structural Health Monitoring. Proceedings of the Optics InfoBase Conference Papers.

